# Broiler Chickens as Source of Human Fluoroquinolone-Resistant *Escherichia coli*, Iceland

**DOI:** 10.3201/eid1601.090243

**Published:** 2010-01

**Authors:** Thorunn R. Thorsteinsdottir, Gunnsteinn Haraldsson, Vala Fridriksdottir, Karl G. Kristinsson, Eggert Gunnarsson

**Affiliations:** University of Iceland, Reykjavík, Iceland (T.R. Thorsteinsdottir, G. Harladsson, V. Fridriksdottir, K.G. Kristinsson, E. Gunnarsson); Landspitali University Hospital, Reykjavík (G. Haraldsson, K.G. Kristinsson)

**Keywords:** Escherichia coli, antimicrobial resistance, fluoroquinolones, bacteria, broiler meat, feed, humans, Iceland, dispatch

## Abstract

To investigate feed as a source for fluoroquinolone-resistant *Escherichia coli* in broiler chickens, we compared antimicrobial drug–resistant *E. coli* from broiler feed and broilers with ciprofloxacin-resistant human clinical isolates by using pulsed-field gel electrophoresis. Feed was implicated as a source for ciprofloxacin-resistant broiler-derived *E. coli* and broilers as a source for ciprofloxacin-resistant human-derived *E. coli*.

In a previous study, we found a relatively high prevalence of antimicrobial and especially quinolone resistance among *Escherichia coli* isolates from broiler chickens and broiler meat (*1*), despite no known antimicrobial drug selection pressure in chicken farming in Iceland and biosecurity measures to prevent transmission of infectious agents into farms. Broiler houses are cleaned and disinfected after broiler flocks are transported to slaughter. Therefore, resistant bacteria are unlikely to persist in the broiler houses. However, animal feed can be contaminated with antimicrobial drug–resistant *E. coli* (*2*).

The high prevalence of quinolone-resistant *E. coli* isolates obtained from broilers and broiler meat coincides with an increasing prevalence of fluoroquinolone resistance among human clinical *E. coli* isolates in Iceland. This increase correlated with increased use of fluoroquinolones in clinical settings (*3*).

We examined whether the prevalence of resistant *E. coli* strains in broilers had changed since our previous study and whether broiler feed could be a source for the resistant strains. Furthermore, we compared the genotypes of ciprofloxacin-resistant broiler, broiler meat, and broiler feed *E. coli* isolates with ciprofloxacin-resistant human clinical *E. coli* isolates.

## The Study

The sampling period for this study was May through November 2008. Pooled cecal samples (20 ceca from each flock) were taken from the 30 flocks slaughtered at all 3 broiler slaughterhouses in Iceland in June 2008. Ceca were stomachered in phosphate-buffered saline, spread on MacConkey agar with and without enrofloxacin (0.25 mg/L), and incubated overnight. Feed was sampled from feed stalls at 18 farms (of which 14 had participated in the previous study) and from 2 feed mills; the feed was suspended in buffered peptone water, mixed, incubated overnight, and spread on MacConkey agar as described above. One colony from each agar plate was selected for susceptibility testing as described in our previous study (*1*). Because isolates were collected from the 18 largest broiler farms (of the 27 farms operating in Iceland), all the broiler slaughterhouses, and the only 2 feed mills operating in Iceland, we believe this study provides a representative sample.

We selected all 34 available human *E. coli* isolates recovered from routine clinical specimens (mostly urine and blood) at the main clinical microbiology/reference laboratory in Iceland (Landspitali University Hospital) during 2006–2007, which had similar susceptibility patterns to the strains previously isolated from broilers (*1*) (ampicillin-tetracycline-sulfamethoxazole/trimethoprim-ciprofloxacin or ciprofloxacin alone). Only 1 isolate was chosen from each patient.

We performed susceptibility testing using a microbroth dilution method (VetMIC; National Veterinary Institute, Uppsala, Sweden). MICs were determined for ampicillin, cefotaxime, ceftiofur, chloramphenicol, ciprofloxacin, florfenicol, gentamicin, nalidixic acid, kanamycin, streptomycin, sulfamethoxazole, tetracycline, and trimethoprim. Cutoff values were those used in the monitoring programs in Sweden and Norway (*4*,*5*). Strains resistant to >3 classes of antimicrobial agents were considered multiresistant.

We compared all *E. coli* broiler ceca and feed isolates resistant to >1 antimicrobial agents and the 34 ciprofloxacin-resistant human *E. coli* isolates with resistant *E. coli* isolates from the previous study (2005–2007) (*1*) by pulsed-field gel electrophoresis (PFGE) using a slightly modified method of Ribot et al. (*6*). Comparison of PFGE patterns was made by visual inspection and BioNumerics software (Applied Maths, Sint-Martens-Latem, Belgium). For cluster analysis, the Dice coefficient for band matching (band-position tolerance 1.5%) was used to generate an unweighted pair group method with arithmetic averages dendrogram. Isolates from the previous and present study that did not yield a satisfactory banding pattern by PFGE were genotyped by randomly amplified polymorphic DNA (RAPD) analysis as described (*7*). Reaction products were analyzed by electrophoresis on 1.5% agarose gels stained with ethidium bromide. Patterns were considered different when the profiles differed by at least 1 band. Similarity among RAPD patterns was compared as described for PFGE. Clusters for PFGE and composite RAPD profiles were defined as >2 isolates with >80% similarity. Prevalence values were compared by using the Fisher exact test.

Of the 40 broiler isolates, 20 were resistant to >1 of the antimicrobial drugs tested (Table); only 1 was multidrug resistant (resistant to streptomycin, tetracyclin, sulfamethoxazole, and trimethoprim). Ciprofloxacin and nalidixic acid were always cross-resistant. Compared with the previous sampling, the prevalence of resistance increased significantly for ciprofloxacin and nalidixic acid (from 18.2% to 42.5%; p<0.0001) but decreased significantly for ampicillin (from 18.2% to 0.0%; p = 0.002) and sulfamethoxazole (from 19.1% to 5.0%; p = 0.0398). This finding suggests that quinolone resistance was not transferred with resistance to the other antimicrobial agents and that it was selected for by other factors. Of the 22 *E. coli* isolates obtained from feed, 7 (32%) were resistant to ciprofloxacin and nalidixic acid, and all were susceptible to the other antimicrobial agents tested. Although no *E. coli* were isolated from the 2 feed mill samples, other *Enterobacteriaceae* grew on the agar plates, which could have overgrown existing *E. coli* strains, if any, demonstrating that the feed was not sterile.

**Table:** Antimicrobial drug resistance among Escherichia coli isolates collected from broiler ceca and feed during 2008 compared with isolates collected from ceca and meat during 2005–2007 (1), Iceland*

Antimicrobial drug	Resistant strains, no. (%)				
	2005–2007			2008	
	Ceca, n = 110	Meat, n = 75		Ceca, n = 40	Feed, n = 22
Ampicillin	20 (18.2)	12 (16.0)		0	0
Cefotaxime	0	0		0	0
Ceftiofur	0	0		0	0
Chloramphenicol	0	0		0	0
Ciprofloxacin	20 (18.2)	27 (36.0)		17 (42.5)	7 (31.8)
Enrofloxacin	16 (14.5)	25 (33.3)		NT	NT
Florfenicol	1 (0.9)	0		0	0
Gentamicin	2 (1.8)	1 (1.3)		0	0
Nalidixic acid	20 (18.2)	27 (36.0)		17 (42.5)	7 (31.8)
Kanamycin	1 (0.9)	0		0	0
Streptomycin	9 (8.2)	7 (9.3)		2 (5.0)	0
Sulfamethoxazole	21 (19.1)	11 (14.7)		2 (5.0)	0
Tetracycline	15 (13.6)	8 (10.7)		1 (2.5)	0
Trimethoprim	16 (14.5)	10 (13.3)		1 (2.5)	0

*NT, not tested. Resistance breakpoints (mg/mL): ampicillin, >16; cefotaxime, >0.5; ceftiofur, >2; chloramphenicol, >32; ciprofloxacin, >0.12; enrofloxacin, >0.5; florfenicol, >32; gentamicin, >8; nalidixic acid, >32; kanamycin, >16; streptomycin, >64; sulfamethoxazole, >512; tetracycline, >16; trimethoprim, >4.

The 27 resistant broiler and feed isolates were compared with 76 resistant isolates analyzed in our previous study (*1*) along with the 34 ciprofloxacin-resistant human *E. coli* isolates. Of 137 broiler, broiler meat, feed and human isolates, 110 (80%) yielded interpretable, reproducible PFGE patterns. We detected 92 profiles, of which 81 (88%) were represented by a single isolate. Isolates of different origin were intermixed forming 26 clusters, of which 12 were seen in the previous study. Of the 14 new clusters, 10 contained isolates of different origins (Figure). Human isolates clustered with broiler (2005–2006), broiler meat, broiler (2008), and feed isolates in 6 clusters (Figure). This supports previous findings of chickens and their products as a possible source of antimicrobial drug–resistant *E. coli* in humans (*8*,*9*). With the extensive genomic diversity of *E. coli* and the discriminative power of PFGE typing, finding indistinguishable isolates of different origin collected over several years is unlikely, except from a large collection (*8*,*10*). Therefore, finding human isolates closely related (>80 similarity) to broiler, broiler meat, and feed isolates suggests an epidemiologic link between the populations. Additionally, we found closely related isolates from feed and broiler (samples from 2008 and 2005–2006) from geographically distant farms, supporting previous findings that antimicrobial drug–resistant *E. coli* could be introduced into the farm environment through broiler feed (*2*).

**Figure:**
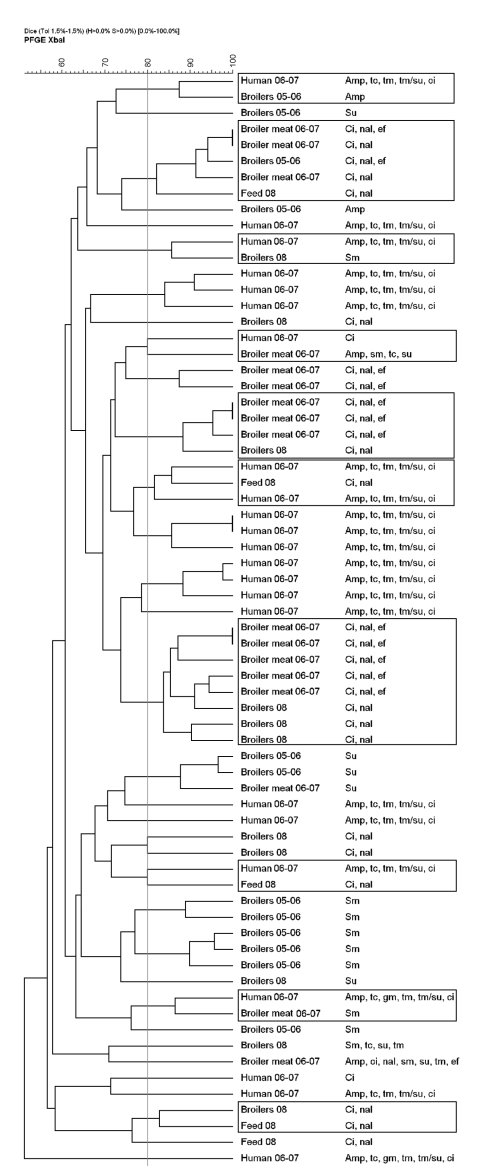
Dendrogram based on unweighted pair group method with arithmetic averages cluster analysis of pulsed-field gel electrophoresis (PFGE) patterns of the 20 broiler (2008), 7 feed (2008), and 34 human ciprofloxacin-resistant *Escherichia coli* isolates along with 29 of the most closely related broiler (2005–2006) and broiler meat (2006–2007) isolates from an earlier study (*1*), Iceland. Boxes indicate clusters (isolates with >80% similarity by Dice coefficient similarity analysis) of isolates of different origins not seen in the previous study. Amp, ampicillin; ci, ciprofloxacin; gm, gentamicin; nal, nalidixic acid; sm, streptomycin; su, sulfamethoxazole; tc, tetracycline; tm, trimethoprim; tm/su, trimethoprim/sulfamethoxazole.

The isolates that did not give interpretable PFGE patterns (1 broiler [2005–2006] and 4 broiler meat [2006–2007] isolates, 11 broiler [2008] and feed [2008] isolates, and 11 ciprofloxacin-resistant human isolates) were subjected to RAPD analysis. All isolates yielded interpretable patterns displaying 26 distinct profiles; all but 1 unique profile represented a single isolate. At 80% similarity, 1 cluster was of mixed origin, containing 6 isolates from feed and broilers (2008).

## Conclusions

Prevalence of fluoroquinolone-resistant *E. coli* remains moderately high in broilers, but resistance to other antimicrobial drugs is decreasing. Fluoroquinolone-resistant *E. coli* isolated from broiler feed implicates feed as the source of resistant strains into farms. Resistant isolates from feed, broilers, broiler meat, and humans were closely related, demonstrating that poultry and their food products can be a source of resistant *E. coli* in humans.
